# Longitudinal Clinical and Molecular Characterization of a Single Pediatric Case of Vertically Acquired HIV-1 Subtype C with Baseline Resistance to Protease Inhibitors

**DOI:** 10.3390/microorganisms14081636

**Published:** 2026-07-27

**Authors:** Kawther A. Zaher, Mai M. El-Daly, Eitezaz A. Zaki, Mohammad M. Alhazmi, Ahmed Abdulhaq, Sherif A. El-Kafrawy, Esam I. Azhar

**Affiliations:** 1 Neuroscience and Geoscience Unit, King Fahd Medical Research Center, King Abdulaziz University, Jeddah 21589, Saudi Arabia; kzaher@kau.edu.sa; 2 Special Infectious Agents Unit-BSL3, King Fahd Medical Research Center, King Abdulaziz University, Jeddah 21589, Saudi Arabia; saelkfrawy@kau.edu.sa (S.A.E.-K.); eazhar@kau.edu.sa (E.I.A.); 3 Department of Medical Laboratory Sciences, Faculty of Applied Medical Sciences, King Abdulaziz University, Jeddah 21589, Saudi Arabia; eitezaz.zaki@gmail.com; 4 Molecular Biology Department, Jeddah Regional Lab, Ministry of Health, Jeddah 22421, Saudi Arabia; 5 Department of Medicine, King Fahad Central Hospital, Ministry of Health, Jazan 84944, Saudi Arabia; alhazmimo@hotmail.com; 6 Deanship of Scientific Affairs and Research, Jazan University, Jazan 45142, Saudi Arabia; alhaq444@gmail.com

**Keywords:** HIV-1 subtype C, pediatric HIV, vertical transmission, transmitted drug resistance, protease inhibitor resistance, integrase inhibitor therapy

## Abstract

Transmitted HIV drug resistance can compromise treatment outcomes in children with vertically acquired infection, particularly when baseline resistance testing is unavailable. This case report describes the longitudinal clinical, virological, immunological, and molecular course of a male child diagnosed with vertically acquired HIV-1 subtype C infection, most consistent with mother-to-child transmission. Clinical history, viral load, CD4/CD8 profiles, HIV-1 pol sequencing, phylogenetic analysis, and interpretation were performed using the Stanford HIV Drug Resistance Database (HIVdb). The baseline viral load was 240,993 copies/mL, with a CD4:CD8 ratio of 0.40. Genotypic analysis identified major protease inhibitor resistance mutations V82A and I84I/V, together with M184V. Stanford HIVdb predicted high-level resistance to lopinavir/ritonavir, atazanavir/ritonavir, lamivudine, and emtricitabine; low-level resistance to darunavir/ritonavir and abacavir; and preserved susceptibility to tenofovir, zidovudine, and the evaluated NNRTIs. Initial lopinavir/ritonavir-based regimens failed to achieve sustained virological suppression. Following treatment optimization with an integrase inhibitor-based regimen and subsequent transition to bictegravir/emtricitabine/tenofovir alafenamide (BIC/FTC/TAF), HIV-1 RNA became Target Not Detected (TND) from November 2024 onward. At the latest follow-up, the CD4 count was 877 cells/µL, the CD8 count was 722 cells/µL, and the CD4:CD8 ratio had improved to 1.21, indicating substantial immune recovery. This single-patient case highlights the clinical value of baseline genotypic resistance testing and individualized, genotype-guided antiretroviral therapy in achieving durable virological suppression in pediatric HIV.

## 1. Introduction

The long-term success of antiretroviral therapy (ART) depends on selecting a regimen fully active against the patient’s circulating virus. This is particularly important in newly diagnosed HIV-1 infection, where pretreatment or transmitted drug resistance may already be present before therapy begins. Although advances in ART have markedly reduced HIV-related morbidity and mortality, drug resistance remains an important obstacle to durable viral suppression [1,2,3,4].

Pediatric HIV infection poses additional clinical challenges. In children with vertically acquired infection, resistant virus may be transmitted from the mother during pregnancy, delivery, or breastfeeding, particularly when maternal infection is undiagnosed, untreated, or inadequately suppressed at the time of transmission [5,6].

Several case reports and clinical studies have described vertically acquired HIV infection complicated by transmitted or acquired antiretroviral drug resistance. Early reports documented vertical transmission of multidrug-resistant HIV-1 from mother to infant and highlighted the potential impact of baseline resistance mutations on subsequent treatment outcomes. Other pediatric cases have documented the emergence of resistance to protease inhibitors and nucleoside reverse transcriptase inhibitors during therapy, emphasizing the importance of early resistance testing and individualized treatment selection. More recently, reports describing integrase strand transfer inhibitor resistance in children have further illustrated the evolving complexity of antiretroviral resistance patterns in pediatric HIV management. Although these reports have substantially improved our understanding of pediatric HIV drug resistance, comprehensive longitudinal case reports integrating baseline genotypic resistance, phylogenetic analysis, immunological recovery, and long-term clinical outcomes remain relatively uncommon, particularly for HIV-1 subtype C infection. Therefore, the present report provides a comprehensive clinical and molecular characterization of the long-term consequences of transmitted drug resistance and genotype-guided treatment optimization in a child with vertically acquired HIV-1 infection [7,8,9].

HIV-1 subtype C is widely distributed globally and has also been documented in Saudi cohorts [2,10,11]. Subtype background and naturally occurring polymorphisms may influence the emergence and interpretation of resistance-associated mutations [12,13]. Major protease inhibitor (PI) resistance mutations, such as V82A and I84V, can reduce susceptibility to several boosted PIs, depending on the accompanying mutational background [13,14]. Curated genotype interpretation systems, such as the Stanford HIV Drug Resistance Database (HIVdb), are widely used to translate observed mutations into drug-specific resistance calls [15,16]. Integrase strand transfer inhibitor (INSTI)-based therapy has become central to modern HIV management because of its potency, tolerability, and high genetic barrier to resistance [4,17].

We hypothesized that baseline transmitted protease inhibitor resistance, identified by genotypic resistance testing, contributed to prolonged virological failure despite protease inhibitor-based therapy, and that subsequent genotype-guided optimization to an integrase inhibitor-based regimen would yield durable virological suppression and immune recovery. This case report was conducted to examine this hypothesis through longitudinal clinical, virological, immunological, and molecular follow-up.

Although transmitted HIV drug resistance has been reported, comprehensive longitudinal studies that integrate baseline genotypic resistance, phylogenetic characterization, immunological recovery, and long-term clinical outcomes in children remain relatively uncommon. The present case goes beyond documenting transmitted resistance to show how baseline resistance mutations informed therapeutic decisions and long-term treatment outcomes over a prolonged follow-up period. By integrating standardized Stanford HIVdb interpretation with virological, immunological, and clinical data, this report illustrates the value of genotype-guided individualized antiretroviral therapy in a child with vertically acquired HIV-1 subtype C infection.

## 2. Materials and Methods

### 2.1. Study Design and Case Selection

This longitudinal clinical and molecular case report describes a pediatric patient selected from a previously studied cohort of treatment-naive HIV-1-infected individuals attending the HIV clinic at King Fahd Central Hospital in Jazan, Saudi Arabia [2].

### 2.2. Ethical Considerations

Ethical approval was obtained from the Biomedical Ethics Unit at King Fahd Central Hospital, Jazan, Saudi Arabia (Form #06). Written informed consent for publication of the patient’s clinical information and anonymized data was obtained from the patient’s parent or legal guardian in accordance with institutional and journal requirements. All identifying information has been removed to protect patient confidentiality.

### 2.3. Clinical Data Collection

Clinical data were extracted from available medical records, including demographic characteristics, presumed route of transmission, parental HIV status, co-infections, antiretroviral therapy history, viral load measurements, CD4 and CD8 cell counts, CD4:CD8 ratio, routine laboratory findings, and longitudinal clinical follow-up.

### 2.4. HIV Serological Testing and Viral-Load Quantification

Plasma was screened with the Genscreen ULTRA HIV Ag-Ab assay. HIV-1 RNA viral load was quantified with the Abbott RealTime HIV-1 assay according to the manufacturer’s instructions.

### 2.5. Viral RNA Extraction and HIV-1 Pol Amplification

Viral RNA was extracted using the MagNA Pure Compact Nucleic Acid Isolation Kit (Roche Diagnostics, Mannheim, Germany) per the manufacturer’s instructions [18]. The HIV-1 pol region was amplified by nested RT-PCR with the QIAGEN OneStep RT-PCR Kit (QIAGEN, Hilden, Germany) on a Veriti 96-well thermal cycler (Applied Biosystems, Foster City, CA, USA) [19,20]. Amplified products were verified by agarose gel electrophoresis prior to sequencing, as previously described for HIV-1 drug-resistance genotyping workflows [2,21].

### 2.6. Sequencing and Phylogenetic Subtype Assignment

Sanger sequencing was performed on the Applied Biosystems 3500 Genetic Analyzer (Applied Biosystems, Foster City, CA, USA). Phylogenetic analysis of the pol gene was conducted in MEGA 11 using the neighbor-joining method after alignment with global HIV-1 reference sequences [22].

### 2.7. Genotypic Drug-Resistance Interpretation

Stanford HIVdb version 10.1 was used to identify mutations associated with PR/RT resistance and to generate drug-specific resistance calls and mutation scores [15,16]. The full HIVdb output provides the curated, rule-based framework that underlies these resistance calls.

### 2.8. Soluble Immune Marker Measurement

Levels of IP-10 (Invitrogen, Carlsbad, CA, USA; Catalog# KAC2361), soluble CD27 (sCD27; Invitrogen, Vienna, Austria; Catalog# BMS286INST), soluble CD80 (sCD80; Invitrogen, Vienna, Austria; Catalog# BMS291INST), and soluble CD4 (sCD4; Abcam, Tokyo, Japan; Catalog# ab234569) were measured in triplicate by ELISA following the manufacturers’ instructions.

### 2.9. Longitudinal Treatment and Outcome Assessment

Treatment history was reviewed chronologically. Virologic failure was defined as persistent detectable viremia, whereas virologic suppression was defined as achieving a target viral load of not detected.

### 2.10. Statistical Analysis

Because this is a single-case report, the analysis was descriptive. No comparative statistical tests or meta-analysis were performed.

## 3. Results

### 3.1. Clinical Background

The patient was a Saudi male child diagnosed with HIV-1 infection, aged five years at the time of the study. At the time of manuscript preparation, the patient was 14 years old, and the infection was considered most consistent with vertical transmission. At baseline, viral load was 240,993 copies/mL, CD4 count was 1148 cells/µL, CD8 count was 2899 cells/µL, and the CD4:CD8 ratio was 0.40. Both parents were HIV-positive, and the mother was diagnosed shortly after the child, suggesting a likely undiagnosed maternal infection during pregnancy or delivery.

The patient completed tuberculosis treatment with isoniazid and rifampicin on 3 August 2017. He subsequently missed follow-up for approximately one year, during which he developed otorrhea, which was successfully treated with amoxicillin–clavulanate. Regular clinical follow-up resumed on 11 November 2021, after which he remained asymptomatic and showed normal growth and development throughout follow-up. Antiretroviral therapy was subsequently optimized, and the patient was ultimately transitioned to bictegravir/emtricitabine/tenofovir alafenamide (BIC/FTC/TAF), which remains his current regimen. HIV-1 RNA has remained undetectable (Target Not Detected, TND) since November 2024. At the latest follow-up, the CD4 count was 877 cells/µL, the CD8 count was 722 cells/µL, and the CD4:CD8 ratio was 1.21. Laboratory investigations showed a creatinine concentration of 42 µmol/L, normal liver function tests, a white blood cell count of 6.13 × 10^9^/L, hemoglobin of 12.7 g/dL, a platelet count of 352 × 10^9^/L, normal C-reactive protein, and an erythrocyte sedimentation rate of 2 mm/h. Hemoglobin electrophoresis confirmed sickle cell trait, with hemoglobin A of 62%, hemoglobin A_2_ of 4%, and hemoglobin S of 34%.

### 3.2. Phylogenetic Analysis

**Figure 1:**
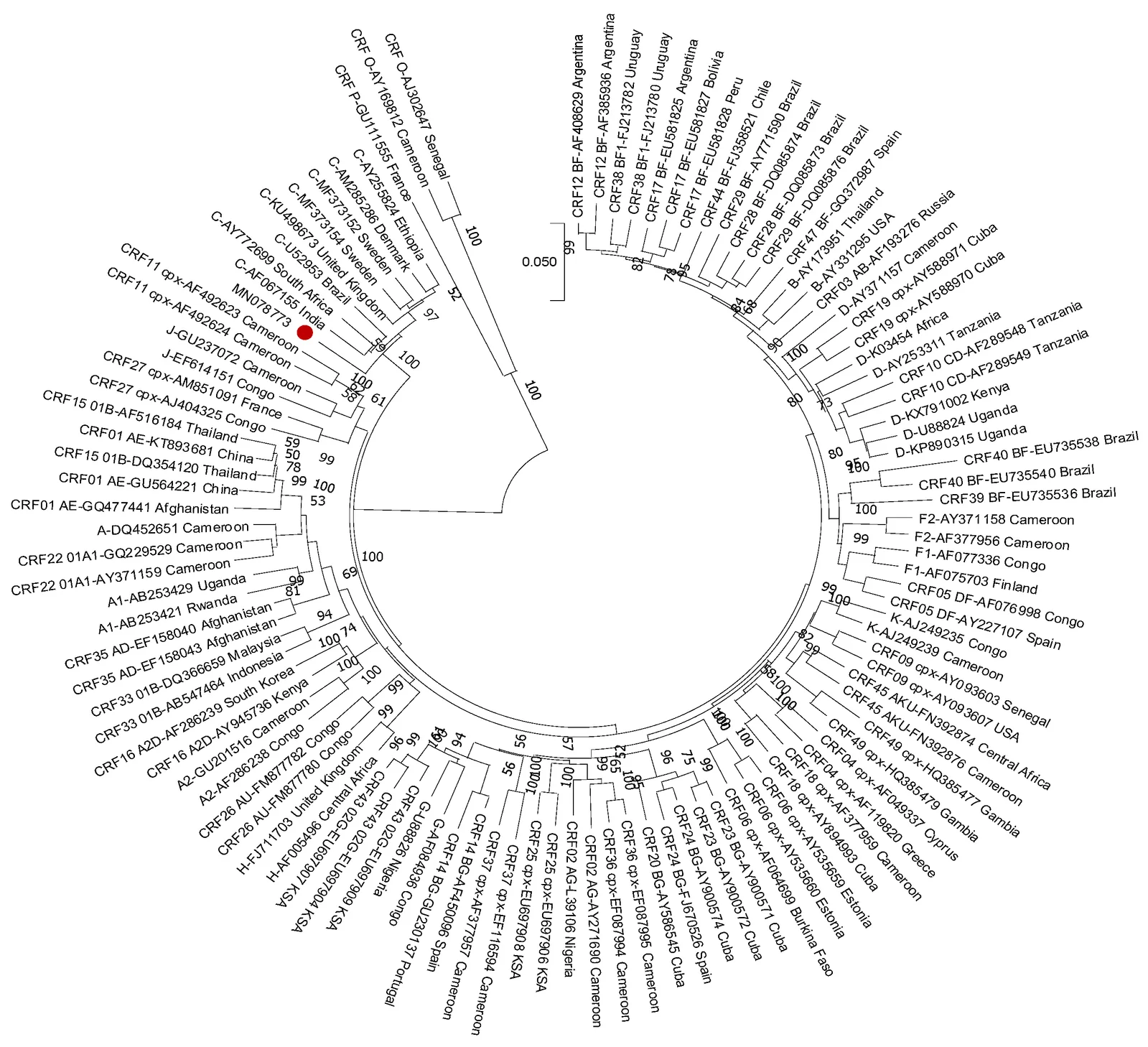
Phylogenetic analysis of the HIV-1 pol sequence (accession number MN078773) was conducted using the neighbor-joining method. A bootstrap consensus tree was constructed from 1000 replicates, and evolutionary distances were computed using the maximum composite likelihood method. All evolutionary analyses were performed in MEGA11. The red dot indicates the study sample, which clusters within the HIV-1 subtype C clade. The scale bar indicates genetic distance.

Phylogenetic analysis of the HIV-1 pol region (accession number MN078773) showed clustering within the HIV-1 subtype C clade (Figure 1).

### 3.3. Drug-Resistance Profile: PI, NRTI, and NNRTI

**Figure 2:**
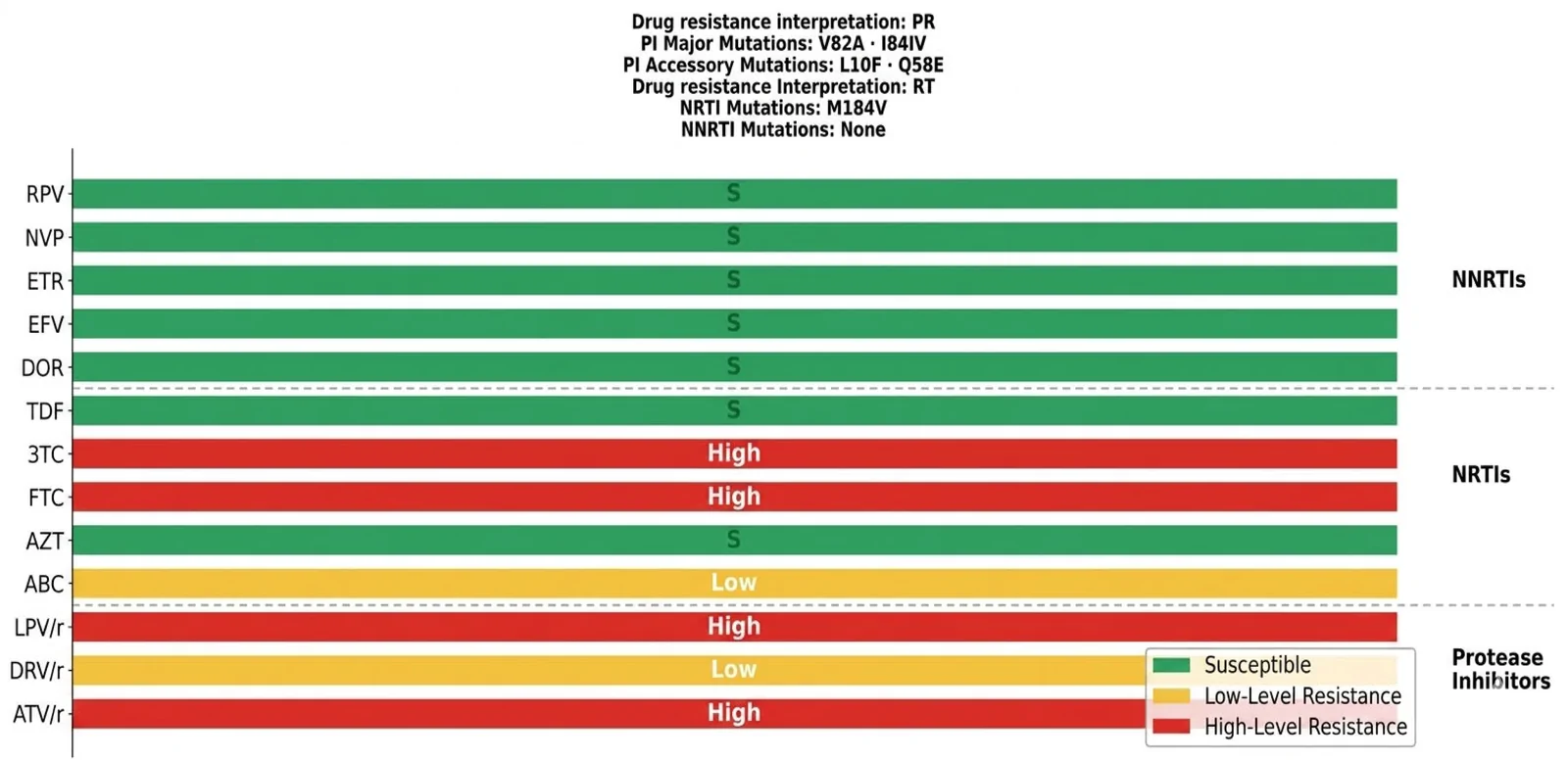
Drug-resistance profile predicted by Stanford HIVdb v10.1. Drug-resistance interpretation: PR; PI major mutations: V82A and I84I/V; PI accessory mutations: L10F and Q58E; RT interpretation: NRTI mutation M184V and no NNRTI mutations. ATV/r, atazanavir/ritonavir; DRV/r, darunavir/ritonavir; LPV/r, lopinavir/ritonavir; ABC, abacavir; AZT, zidovudine; FTC, emtricitabine; 3TC, lamivudine; TDF, tenofovir; DOR, doravirine; EFV, efavirenz; ETR, etravirine; NVP, nevirapine; RPV, rilpivirine.

Stanford HIVdb version 10.1 identified the major PR mutations V82A and I84I/V, along with the accessory PR mutations L10F and Q58E, and the major NRTI mutation M184V. No NNRTI mutations were detected. The resulting drug-resistance predictions across three drug classes are shown in Figure 2. The resistance profile indicated high-level resistance to ATV/r and LPV/r, low-level resistance to DRV/r, high-level resistance to 3TC and FTC, low-level resistance to ABC, susceptibility to AZT and TDF, and maintained susceptibility to all five evaluated NNRTIs.

### 3.4. Stanford HIVdb Sequence Analysis Report

**Figure 3:**
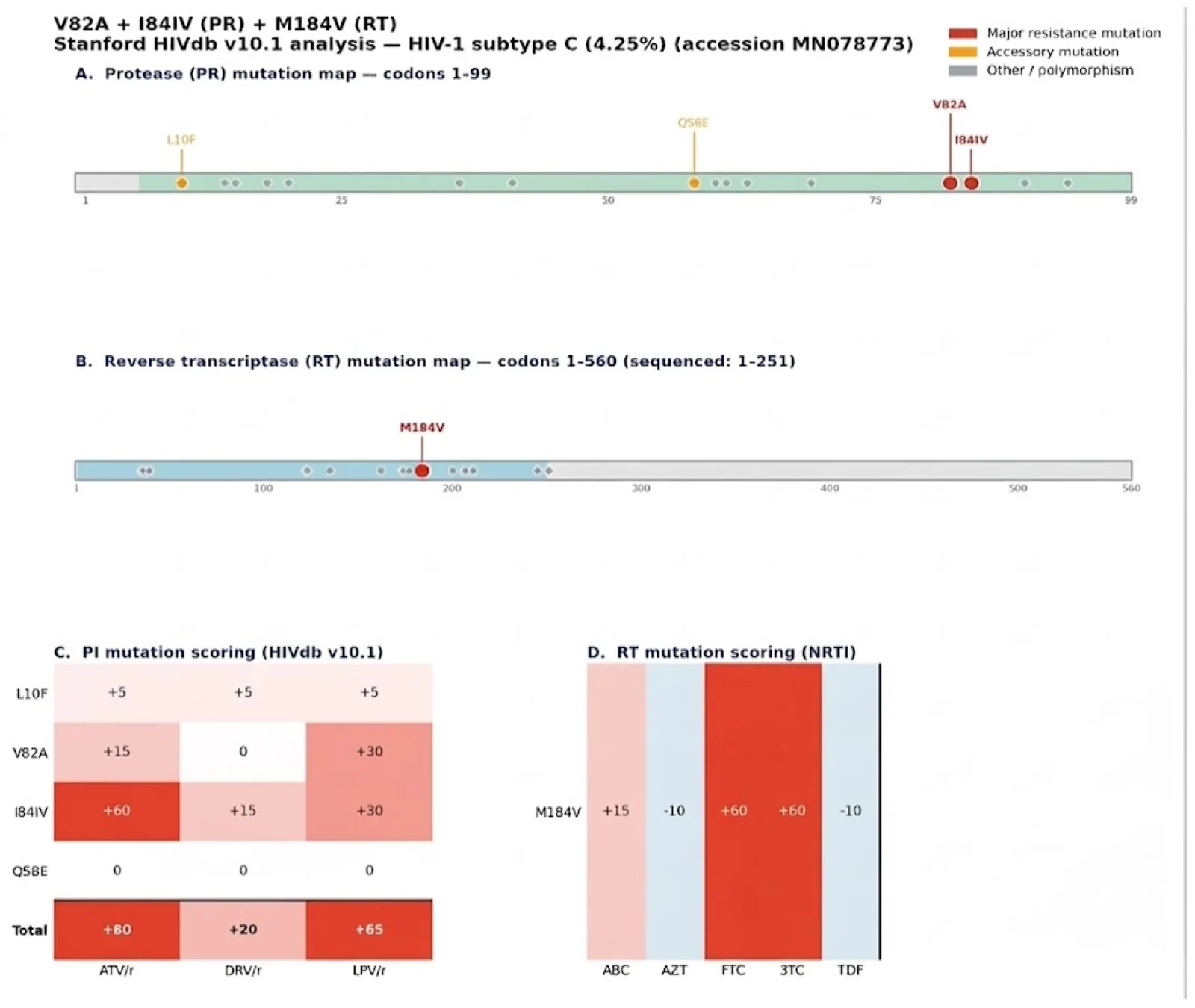
Stanford HIVdb v10.1 drug-resistance analysis. Panel (**A**): protease (PR) mutation-position map (codons 1–99) showing major and accessory substitutions detected in the sequenced region. Panel (**B**): reverse-transcriptase (RT) mutation map (codons 1–560; sequenced region 1–251). Panels (**C**,**D**): mutation scoring for PI- and RT-associated resistance.

The complete Stanford HIVdb v10.1 sequence analysis report for this case is shown in Figure 3. The report summarizes the sequenced PR codons 6–99 and RT codons 1–251, highlights the significant drug-resistance mutations V82A and I84I/V in protease and M184V in reverse transcriptase, and provides mutation-level scoring and drug-specific resistance calls. According to the Stanford HIV Drug Resistance Database (HIVdb) interpretation algorithm [16], the interpretive panel also indicates that darunavir should be administered twice daily if prescribed in this mutational context [15].

### 3.5. Soluble Immune Marker Profile

**Figure 4:**
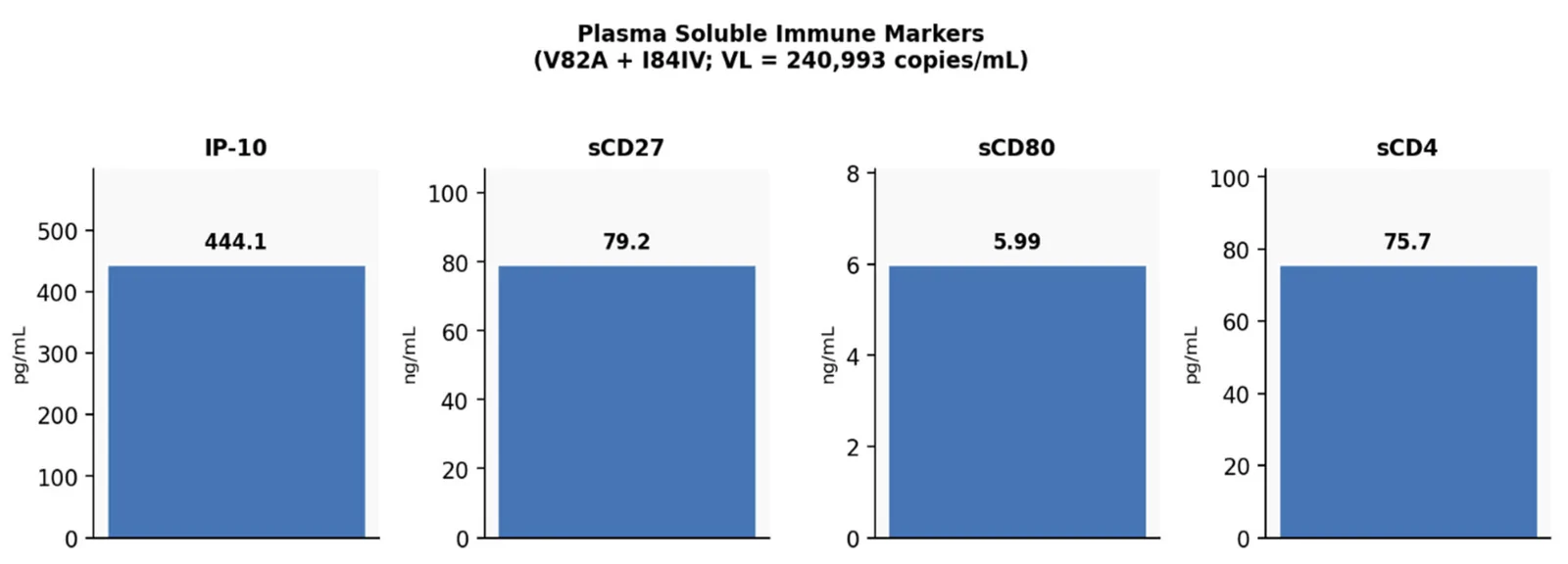
Baseline plasma soluble immune marker profile of the study patient. Bar charts show the concentrations of interferon gamma-induced protein 10 (IP-10), soluble CD27 (sCD27), soluble CD80 (sCD80), and soluble CD4 (sCD4) measured in plasma before optimization of antiretroviral therapy. Elevated IP-10 and sCD27 levels are consistent with persistent immune activation associated with uncontrolled HIV-1 infection, whereas the concentrations of sCD80 and sCD4 are shown for comparison. Marker concentrations are presented in their respective units as indicated on the y-axes.

At baseline, plasma levels of soluble immune markers were as follows: IP-10, 444.1 pg/mL; sCD27, 79.2 ng/mL; sCD80, 5.99 ng/mL; and sCD4, 75.7 pg/mL (Figure 4). The elevated IP-10 level is consistent with interferon-driven inflammation in untreated HIV, whereas increased sCD27 and sCD80 levels are consistent with heightened immune activation during high-level viremia.

### 3.6. Longitudinal Treatment History and Virological Outcomes

Initial ART, started in November 2015, consisted of LPV/r (80/20 mg), AZT, and 3TC. Virologic suppression was not achieved, with viral loads of 112,369 copies/mL in 2016 and 9204 copies/mL in November 2020. In April 2021, the regimen was modified to TDF/FTC combined with LPV/r. Despite this change, the viral load remained detectable at 2840 copies/mL in August 2022.

At the latest follow-up, the patient remained clinically stable and asymptomatic with sustained virological suppression and continued immune recovery. Laboratory investigations remained stable and are summarized in Table 1.

At the latest follow-up, the patient remained clinically stable and asymptomatic, with sustained virological suppression and ongoing immunological recovery. Laboratory testing showed a CD4 count of 877 cells/µL, a CD8 count of 722 cells/µL, and a CD4:CD8 ratio of 1.21. Renal and hepatic function remained within normal limits (serum creatinine, 42 µmol/L; normal liver function tests). Hematological parameters were also within normal limits, with a white blood cell count of 6.13 × 10^9^/L, hemoglobin of 12.7 g/dL, and a platelet count of 352 × 10^9^/L. Inflammatory markers were unremarkable, with a normal C-reactive protein level and an erythrocyte sedimentation rate of 2 mm/h. Hemoglobin electrophoresis identified sickle cell trait (HbA 62%, HbA_2_ 4%, HbS 34%), without associated clinical complications during follow-up.

## 4. Discussion

### 4.1. Pediatric Vertical Transmission and Pretreatment Resistance

This longitudinal case report demonstrates that baseline protease inhibitor resistance, together with M184V-associated NRTI resistance, can compromise early antiretroviral treatment in vertically acquired pediatric HIV-1 infection when resistance testing is unavailable or ignored. By linking the baseline genotype to treatment history, persistent viremia, subsequent integrase inhibitor-based optimization, target-not-detected viral load, and immune recovery, this case provides a clinically relevant example of the value of genotype-guided ART selection. The findings support early HIV-1 genotypic resistance testing in pediatric infection, particularly when vertical transmission is suspected, and maternal treatment history is incomplete. They also emphasize that resistance results should be interpreted within the broader clinical trajectory to guide timely selection of a fully active regimen. The identification of major PI resistance mutations (V82A + I84I/V) in a treatment-naive child strongly suggests vertical transmission rather than resistance selected by the child’s own treatment. Vertically transmitted HIV resistance in children is well recognized but may be underreported when maternal infection is undiagnosed or maternal ART is initiated late [5,6]. In the present case, the mother was diagnosed only after the child, supporting the likelihood of untreated maternal infection during pregnancy and delivery.

### 4.2. Stanford HIVdb-Based Interpretation as Reference Framework

The interpretive framework used in this study was Stanford HIVdb v10.1 (Figure 3). HIVdb provides a standardized, curated system for translating mutation patterns into drug-specific resistance calls [15,16]. V82A is a major PI resistance mutation selected by PI exposure and is associated with reduced susceptibility to several PIs, particularly when combined with other major PI mutations. I84V reduces susceptibility to LPV/r, ATV/r, and DRV/r, whereas M184V confers high-level resistance to 3TC and FTC, reduces viral replicative fitness, and increases susceptibility to some thymidine-analog or tenofovir-containing backbones [13,14,23,24,25].

### 4.3. ART Trajectory and Virologic Outcomes

**Figure 5:**
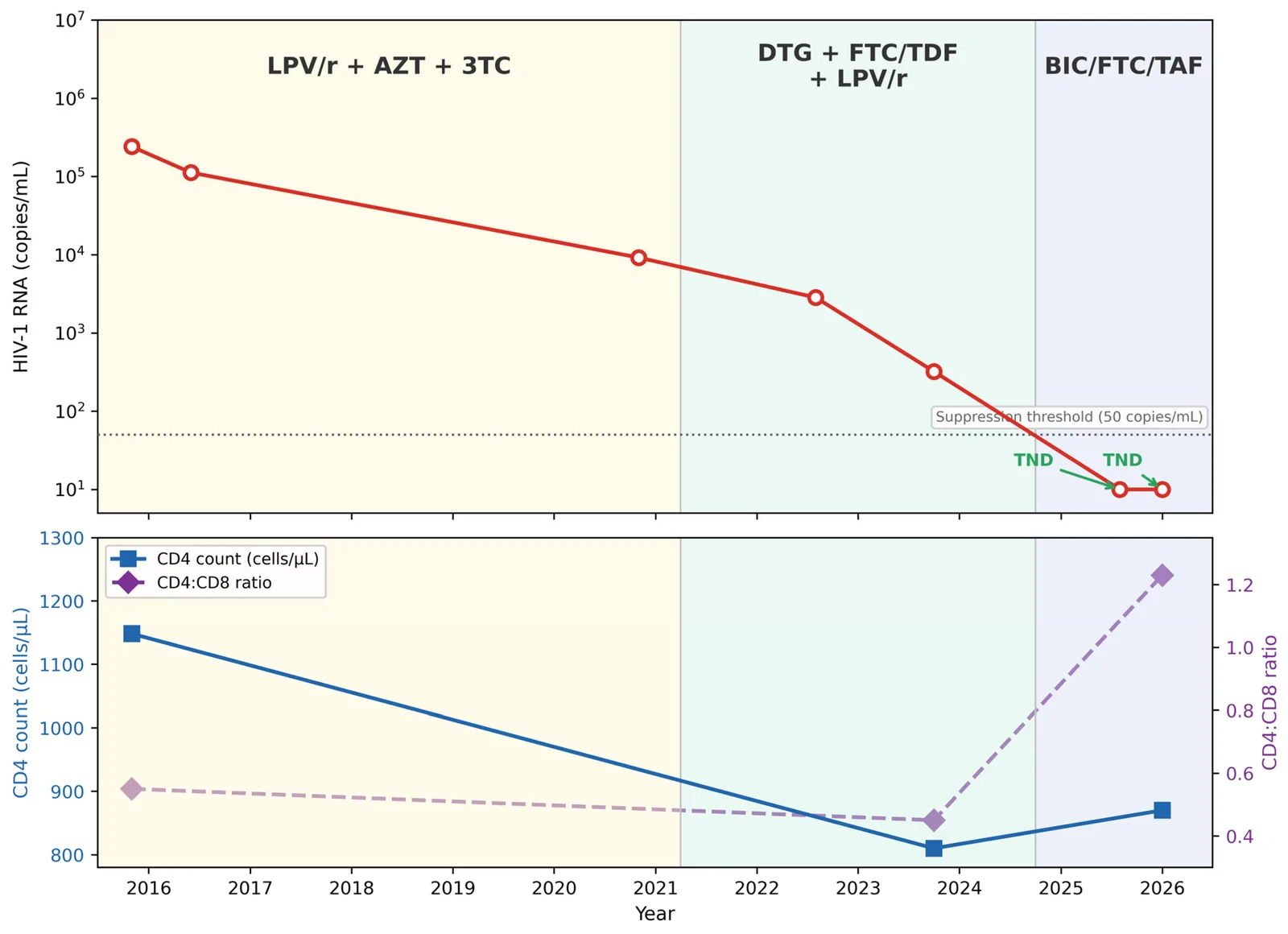
Longitudinal virological and immunological response during antiretroviral therapy. The upper panel shows HIV-1 RNA levels over time on a logarithmic scale. The dashed horizontal line indicates the virological suppression threshold (50 copies/mL), The red line/circles (HIV-1 RNA viral load), the grey dotted line (suppression threshold at 50 copies/mL), and the green arrows/text (TND, Target Not Detected). The lower panel illustrates longitudinal changes in CD4 count and CD4:CD8 ratio. Background shading represents successive antiretroviral treatment regimens.

The longitudinal clinical course illustrates the consequences of initiating ART in the presence of baseline PI resistance. The initial LPV/r-based therapy was predicted to be ineffective because of high-level LPV/r resistance conferred by V82A + I84I/V (Figure 2 and Figure 3), which explains the sustained virologic failure observed from 2015 to 2022 (Figure 5). In 2023, switching to a dolutegravir-containing regimen resulted in partial viral suppression. Subsequent introduction of bictegravir/TAF/FTC in October 2024 achieved complete viral suppression by August 2025, which was maintained through January 2026. This outcome supports the current preference for INSTI-based regimens when clinically appropriate [4,17].

The principal contribution of this report is not merely the identification of transmitted drug resistance, which has been previously described, but rather the comprehensive longitudinal evaluation of the clinical consequences of that resistance. By integrating baseline molecular characterization, phylogenetic analysis, serial virological and immunological assessments, and long-term clinical follow-up, this case provides a detailed illustration of how genotype-guided optimization of antiretroviral therapy led to sustained virological suppression and immune recovery in a child with vertically acquired HIV-1 subtype C infection.

### 4.4. Immune Reconstitution Following Virologic Suppression

At the latest follow-up, the patient maintained durable virological suppression, with HIV-1 RNA remaining Target Not Detected (TND). The CD4 count was 877 cells/µL, the CD8 count was 722 cells/µL, and the CD4:CD8 ratio improved to 1.21, indicating substantial immune reconstitution following optimization of antiretroviral therapy (Figure 5). Together with the elevated IP-10 level (Figure 4), these findings are consistent with heightened immune activation in untreated or incompletely suppressed HIV infection [26,27,28,29].

### 4.5. M184V and the NRTI Backbone

The M184V mutation confers high-level resistance to 3TC and FTC but also imposes a viral fitness cost and may increase susceptibility to AZT and TDF [23,24,25]. In the HIVdb scoring output (Figure 3), M184V contributes to high resistance scores for FTC and 3TC and yields negative scores for AZT and TDF. If baseline genotyping had been available in 2015, it would likely have supported earlier avoidance of a compromised PI-based regimen and earlier use of a fully active INSTI-containing regimen.

### 4.6. Maternal ART and the Transmission Context

The mother’s first available viral-load result in April 2017 was already target not detected while on elvitegravir/cobicistat/TAF/FTC. Her regimen was later optimized to DTG/TAF/FTC and BIC/TAF/FTC. The likelihood that she was undiagnosed at delivery underscores the importance of antenatal HIV screening, early maternal ART, and prevention of mother-to-child transmission [4,5].

### 4.7. Comparison with Previously Reported Pediatric Cases

The present case should be considered in the context of previously reported pediatric HIV drug-resistance cases. Vertical transmission of drug-resistant HIV has been recognized for more than two decades. Johnson et al. first described vertical transmission of multidrug-resistant HIV-1, followed by continued evolution of resistance in an infected infant, highlighting that resistant viral strains can be transmitted from mother to child and subsequently limit therapeutic options. Likewise, previous pediatric studies have shown that transmitted or pretreatment resistance may compromise the effectiveness of first-line antiretroviral therapy, emphasizing the importance of baseline resistance testing before treatment initiation [5,6,7].

The baseline resistance profile observed in our patient, including the major protease inhibitor mutations V82A and I84I/V, together with the M184V reverse-transcriptase mutation, was highly consistent with the prolonged virological failure during the initial protease inhibitor-based regimen. This observation aligns with the Stanford HIV Drug Resistance Database interpretation and with previous studies demonstrating that combinations of major protease inhibitor resistance mutations markedly reduce susceptibility to boosted protease inhibitors, while M184V confers high-level resistance to lamivudine and emtricitabine [13,14,15,16,17,23,27,28,29].

More recently, O’Connell et al. reported the rapid development of drug resistance during initial dolutegravir-based therapy in an infant with HIV [8]. In contrast, integrase resistance testing was not performed in our patient because sustained virological suppression was ultimately achieved, so no conclusion can be drawn about the presence or absence of integrase inhibitor resistance. Furthermore, the incomplete virological response observed during the earlier treatment phase cannot be attributed solely to dolutegravir. Multiple factors, including adherence, background regimen activity, pharmacokinetics, duration of therapy, and the cumulative impact of pre-existing resistance mutations, may have contributed to the observed clinical course. Consequently, the successful suppression achieved after switching to bictegravir/emtricitabine/tenofovir alafenamide should be interpreted as the result of overall genotype-guided regimen optimization rather than as evidence of the superiority of one integrase inhibitor over another.

A recent systematic review and meta-analysis by Fokam et al. confirmed that resistance to nucleoside reverse transcriptase inhibitors, protease inhibitors, and, increasingly, integrase strand transfer inhibitors remains a major challenge in pediatric HIV management [9]. Our findings align with this broader body of evidence and extend previous reports by integrating baseline genotypic resistance testing, phylogenetic subtype assignment, longitudinal virological monitoring, immunological recovery, and long-term clinical follow-up in a child with vertically acquired HIV-1 subtype C infection. Although conclusions cannot be generalized from a single patient, this case illustrates how early resistance testing and individualized treatment selection can substantially improve long-term clinical outcomes [4,9,13,15,16,17].

The findings of this case support our proposed clinical hypothesis. The baseline resistance profile predicted reduced susceptibility to the initial protease inhibitor-based regimen, consistent with the prolonged period of incomplete virological suppression observed during follow-up. Subsequent optimization of antiretroviral therapy based on the resistance profile was associated with durable virological suppression and progressive immune recovery, supporting the clinical utility of genotype-guided treatment selection in pediatric HIV infection. Although conclusions cannot be generalized from a single case, the longitudinal observations provide clinically relevant evidence supporting the proposed hypothesis.

## 5. Conclusions

Beyond documenting transmitted protease inhibitor resistance, this case offers a comprehensive longitudinal view of how baseline genotypic resistance shaped treatment response and how genotype-guided optimization ultimately achieved durable virological suppression and immune recovery. Stanford HIVdb v10.1 provided standardized molecular interpretation, and the longitudinal clinical trajectory confirmed the predicted clinical consequences. Bictegravir/TAF/FTC achieved complete virologic suppression and substantial immune reconstitution. Overall, the longitudinal clinical findings support the hypothesis that baseline genotypic resistance testing can identify transmitted resistance that influences long-term therapeutic outcomes and can facilitate individualized treatment strategies leading to sustained virological suppression and immune recovery. These findings support baseline genotypic resistance testing as an important component of pediatric HIV management.

## 6. Limitation

This report describes the clinical course of a single patient, so the findings should be interpreted with caution and should not be generalized to all children with vertically acquired HIV infection or to those with transmitted HIV drug resistance. Individual variability in viral genetics, treatment history, adherence, host immune responses, and clinical management may influence treatment outcomes. In addition, phenotypic resistance testing was not available, and the maternal antiretroviral treatment history before HIV diagnosis could not be fully reconstructed, limiting definitive confirmation of the origin of the transmitted resistance mutations. Although the patient achieved complete virological suppression after optimization to a bictegravir-based regimen, integrase resistance testing was not performed. Therefore, the relative contribution of viral, pharmacological, or adherence-related factors to the differing virological responses observed during treatment cannot be determined. Future longitudinal studies incorporating integrase resistance testing in pediatric patients with incomplete virological suppression may provide further insight into these mechanisms. Nevertheless, the strength of this case lies in the integration of longitudinal clinical follow-up, virological, immunological, phylogenetic, and genotypic resistance data over an extended observation period, providing a comprehensive illustration of how baseline transmitted resistance influenced treatment response and how genotype-guided optimization ultimately resulted in sustained virological suppression and immune recovery.

## Figures and Tables

**Table 1:** Longitudinal treatment history, clinical events, and virological/immunological outcomes.

Time Point	ART Regimen	Dose	Major Clinical Events	HIV-1 RNA (Copies/mL)	CD4 (Cells/µL)	CD8 (Cells/µL)	CD4:CD8 Ratio
12 November 2015	LPV/r + AZT + 3TC	LPV/r 110 mg every 12 h; AZT 80 mg twice daily; 3TC 35 mg twice daily	Initial HIV diagnosis	240,993	1148	2899	0.40
2016	Same regimen	Weight-adjusted pediatric doses	Persistent viremia	112,369	—	—	—
3 August 2017	Continued ART	—	Completed anti-tuberculosis treatment (isoniazid + rifampicin)	—	—	—	—
2018	Continued ART	—	Lost to follow-up for approximately one year	—	—	—	—
After return	Continued ART	—	Ear discharge treated successfully with amoxicillin/clavulanate (Augmentin)	—	—	—	—
November 2020	Continued ART	Weight-adjusted pediatric doses	Persistent viremia	9204	—	—	—
12 June 2023	AZT + 3TC	AZT 250 mg twice daily; 3TC 75 mg morning/150 mg evening	ART regimen modified	—	—	—	—
2 October 2023	DTG + FTC/TDF + LPV/r	DTG 30 mg once daily; FTC/TDF 120 mg once daily; LPV/r 200 mg twice daily	Integrase inhibitor introduced	322	808	—	0.35
15 October 2024	BIC/FTC/TAF	One tablet (50/200/25 mg) once daily	Switched to bictegravir-based single-tablet regimen	—	—	—	—
November 2024	BIC/FTC/TAF	Same	HIV-1 RNA became Target Not Detected (TND)	TND	—	—	—
Latest follow-up	BIC/FTC/TAF	Same	Clinically asymptomatic; normal growth and development; normal renal and liver function; CRP normal; ESR 2 mm/h; sickle cell trait	TND	877	722	1.21

Abbreviations: LPV/r, lopinavir/ritonavir; AZT, zidovudine; 3TC, lamivudine; DTG, dolutegravir; FTC, emtricitabine; TDF, tenofovir disoproxil fumarate; BIC, bictegravir; TAF, tenofovir alafenamide; TND, target not detected. A complete chronological record of all prescribed medications, dosage adjustments, formulations, routes of administration, treatment durations, and concomitant therapies is provided in Supplementary Table S1.

## Data Availability

The original contributions presented in this study are included in the article/Supplementary Material. Further inquiries can be directed to the corresponding author.

## Supplementary Materials

The following supporting information can be downloaded at: https://www.mdpi.com/article/10.3390/microorganisms14081636/s1, Table S1: Complete chronological medication history, including dosage adjustments, formulations, routes of administration, treatment duration, and concomitant medications during follow-up.

## Abbreviations

The following abbreviations are used in this manuscript:

3TC	Lamivudine
ABC	Abacavir
ART	Antiretroviral therapy
ATV/r	Atazanavir/ritonavir
AZT	Zidovudine
BIC	Bictegravir
BIC/TAF/FTC	Bictegravir/tenofovir alafenamide/emtricitabine
CD4	Cluster of differentiation 4
CD8	Cluster of differentiation 8
DOR	Doravirine
DRV/r	Darunavir/ritonavir
DTG	Dolutegravir
EFV	Efavirenz
ELISA	Enzyme-linked immunosorbent assay
ETR	Etravirine
FTC	Emtricitabine
HIV	Human immunodeficiency virus
HIV-1	Human immunodeficiency virus type 1
HIVdb	Stanford HIV Drug Resistance Database
INSTI	Integrase strand transfer inhibitor
IP-10	Interferon gamma-induced protein 10
LPV/r	Lopinavir/ritonavir
MEGA	Molecular Evolutionary Genetics Analysis
NNRTI	Non-nucleoside reverse transcriptase inhibitor
NRTI	Nucleoside/nucleotide reverse transcriptase inhibitor
NVP	Nevirapine
PI	Protease inhibitor
PR	Protease
RPV	Rilpivirine
RT	Reverse transcriptase
RT-PCR	Reverse-transcription polymerase chain reaction
sCD4	Soluble CD4
sCD27	Soluble CD27
sCD80	Soluble CD80
SDRM	Surveillance drug-resistance mutation
TAF	Tenofovir alafenamide
TDF	Tenofovir disoproxil fumarate
TND	Target not detected
VL	Viral load
