# Supplementary material for: Longitudinal Clinical and Molecular Characterization of a Single Pediatric Case of Vertically Acquired HIV-1 Subtype C with Baseline Resistance to Protease Inhibitors
Source: Microorganisms. 2026 Jul 27;14(8):1636. doi: 10.3390/microorganisms14081636 (PMC13515765; doi:10.3390/microorganisms14081636)
Supplement: Supplementary file 1 [file microorganisms-14-01636-s001.zip › microorganisms-4400178-supplementary.pdf]

Supplementary Table S1. Complete chronological medication history, including dosage adjustments, formulations, routes of administration, treatment duration, and concomitant medications during follow-up.

Date Initiated	Medication	Formulation	Dose	Frequency	Duration	Clinical Purpose
12 Nov 2015	Oseltamivir (OST)	Capsule	30 mg	Twice daily	5 days	Influenza treatment
12 Nov 2015	Lamivudine (3TC)	Oral solution (10 mg/mL)	35 mg	Twice daily	6 months	Initial ART
12 Nov 2015	Zidovudine (AZT)	Syrup (10 mg/mL)	80 mg	Twice daily	6 months	Initial ART
12 Nov 2015	Lopinavir/ritonavir (LPV/r)	Syrup (80/20 mg)	110 mg	Every 12 H	7 months	Initial ART
12 Jun 2023	Zidovudine (AZT)	Tablet	250 mg	Twice daily	12 months	Weight-adjusted regimen
12 Jun 2023	Lamivudine (3TC)	Tablet	75 mg AM /150 mg PM	Daily	12 months	Weight-adjusted regimen
19 Sep 2023	Lamivudine (3TC)	Tablet	220 mg	Every 12 H	12 months	Dose adjustment
19 Sep 2023	Zidovudine (AZT)	Tablet	190 mg	Twice daily	12 months	Dose adjustment
2 Oct 2023	Emtricitabine/Tenofovir (FTC/TDF)	Tablet	120 mg	Once daily	12 months	Backbone regimen
2 Oct 2023	Dolutegravir (DTG)	Tablet	30 mg	Once daily	12 months	Integrase inhibitor
2 Oct 2023	Lopinavir/ritonavir (LPV/r)	Tablet (200/50 mg)	200 mg	Twice daily	12 months	Continued PI therapy
11 Dec 2023	Ferrous sulfate (FS)	Tablet	100 mg	Every 12 H	1 month	Iron supplementation

15 Oct 2024	Bictegravir/Emtricitabine/Tenofovir alafenamide (BIC/FTC/TAF)	Tablet	50/200/25 mg	Once daily	12 months	Optimized single-tablet regimen
2 Dec 2025	Bictegravir/Emtricitabine/Tenofovir alafenamide (BIC/FTC/TAF)	Tablet	50/200/25 mg	Once daily	Ongoing	Optimized single-tablet regimen
